# Hepatobiliary malignancies have distinct peripheral myeloid-derived suppressor cell signatures and tumor myeloid cell profiles

**DOI:** 10.1038/s41598-020-75881-1

**Published:** 2020-11-02

**Authors:** Defne Bayik, Adam J. Lauko, Gustavo A. Roversi, Emily Serbinowski, Lou-Anne Acevedo-Moreno, Christopher Lanigan, Mushfig Orujov, Alice Lo, Tyler J. Alban, Adam Kim, Daniel J. Silver, Laura E. Nagy, J. Mark Brown, Daniela S. Allende, Federico N. Aucejo, Justin D. Lathia

**Affiliations:** 1 Lerner Research Institute, Cleveland Clinic, 9500 Euclid Ave., NE3-202, Cleveland, OH 44195 USA; 2 Case Comprehensive Cancer Center, Cleveland, OH 44106 USA; 3 Cleveland Clinic Lerner College of Medicine at Case Western Reserve University, Cleveland, USA; 4 Department of Pathology, Case Western Reserve University, Cleveland, OH 44106 USA; 5 Department of General Surgery, Cleveland Clinic, 9500 Euclid Ave, Cleveland, OH 44195 USA; 6 Department of Pathology, Cleveland Clinic, Cleveland, OH 44195 USA; 7 Department of Pathological Anatomy, Azerbaijan Medical University, 1022 Baku, Azerbaijan; 8 Case Western Reserve University, Cleveland, OH 44195 USA; 9 Department of Inflammation and Immunity, Northern Ohio Alcohol Center, Center for Liver Disease Research, Cleveland Clinic, Lerner Research Institute, Cleveland, OH 44195 USA; 10 Liver Cancer Center of Excellence, Cleveland Clinic, Cleveland, OH 44195 USA

**Keywords:** Gastrointestinal cancer, Tumour biomarkers, Tumour immunology

## Abstract

Myeloid-derived suppressor cells (MDSCs) are immunosuppressive cells that are increased in patients with numerous malignancies including viral-derived hepatocellular carcinoma (HCC). Here, we report an elevation of MDSCs in the peripheral blood of patients with other hepatobiliary malignancies including non-viral HCC, neuroendocrine tumors (NET), and colorectal carcinoma with liver metastases (CRLM), but not cholangiocarcinoma (CCA). The investigation of myeloid cell infiltration in HCC, NET and intrahepatic CCA tumors further established that the frequency of antigen-presenting cells was limited compared to benign lesions, suggesting that primary and metastatic hepatobiliary cancers have distinct peripheral and tumoral myeloid signatures. Bioinformatics analysis of The Cancer Genome Atlas dataset demonstrated that a high MDSC score in HCC patients is associated with poor disease outcome. Given our observation that MDSCs are increased in non-CCA malignant liver cancers, these cells may represent suitable targets for effective immunotherapy approaches.

## Introduction

Primary hepatocellular carcinoma (HCC) is among the leading cause of cancer-related deaths in the U.S., with 40,000 new cases and 30,000 mortalities in 2018^1^. Chronic hepatitis B virus (HBV) and hepatitis C virus (HCV) infection, obesity, and excess alcohol consumption are risk factors associated with HCC^2^. HCC resulting from chronic viral infections constitutes 75% of all cases, while the non-viral causes account for the remaining 25%. Secondary liver cancers that originate the metastatic spread of a primary tumor from a distant site, most commonly colorectal carcinoma (CRLM), are more frequent than primary HCC^3^. Cholangiocarcinoma (CCA) and neuroendocrine tumors (NETs) are rare hepatobiliary cancers that have unique disease presentation compared to HCC and CRLM and distinct therapeutic responsiveness^4,5^. Current treatment strategies including surgical resection, radiation, ablation, embolization, systemic/local chemotherapy infusion and liver transplantation have improved the outcome of patients with HCC and CCA; however, liver cancers are often diagnosed at an advanced stage with poor clinical presentation and have high recurrence rates^6^. Thus, there is a need to gain mechanistic insight into the pathobiology of liver cancers and develop more effective therapeutic opportunities.

Tumors employ multiple mechanisms to evade immune recognition. Myeloid-derived suppressor cells (MDSCs) are a heterogenous population of immature myeloid cells that expand in patients with malignancies and infiltrate tumors, where they dampen the anti-tumor immune response by suppressing cytotoxic T cell and natural killer (NK) cell activity^7^. Previous work demonstrated that a high MDSC frequency in melanoma not only correlates with poor patient outcome but also associates with a poor response to therapies^7–10^. In multiple preclinical models including colorectal and liver cancers, targeting MDSCs activated an anti-tumor immune response and reduced tumor growth, suggesting that modulating these cells is a promising strategy for cancer immunotherapy^11,12^. Several studies have also reported that MDSCs are increased in the peripheral blood of HCC patients^13–16^. However, the relevance of this observation in terms of other hepatobiliary malignancies, including non-viral HCC, has yet to be determined. Considering the range and pathobiological variation of primary and metastatic liver cancers, we hypothesized that MDSCs might have distinct associations with different types of hepatobiliary tumors. By screening blood and liver surgical specimens from patients with HCC, CCA, CRLM and NET, we determined that MDSCs are elevated in most but not all liver cancers. Our findings demonstrate that the frequency of MDSCs depends on the type of liver tumor and support the screening of MDSCs as a biomarker in patients with HCC, CRLM and NET.

## Results

### Patient characteristics

To gain a more comprehensive understanding of the link between MDSCs and liver cancers, we analyzed MDSC frequency from peripheral blood mononuclear cells (PBMCs) of 114 patients with diagnoses including benign lesion, HCC, CRLM, NET and CCA and 19 healthy controls. The characteristics of these patients are summarized in Table 1. Briefly, 19 patients were diagnosed with HCC, grade 1–3 and TNM stage 1–3. Of the 9 HCC patients with cirrhosis, only one had a Class B Child–Pugh Score (remainder Class A). The benign lesions were primarily hepatic adenomas, with two adrenocortical adenoma, two focal nodular hyperplasia, one biliary cystadenoma, three hemangiomas, two biliary cystadenomas, one pseudolipoma of Glisson’s capsule, and one fibrotic cyst, and one patient had polycystic liver disease. No CRLM patients had cirrhosis, and the intrahepatic lesion number ranged from 1–13. The tumors of the CCA patients ranged from TNM stage 1–4 and grade 1–3, and all but one patient had intrahepatic CCA. Ten out of 22 CCA patients had vascular invasion. The NET ranged from grade 1–3 and included primary tumors in the following locations: small bowel (2), terminal ileum, pancreas, stomach, adrenal cortex (2), large bowel and unknown primary (2). HCC and CCA patients were older than both patients with benign tumors and healthy controls (median age 71.4/67.5/47.6/46.0, respectively, p < 0.0001) and more likely to be male compared to patients with benign tumors (p < 0.01). HCC patients also presented elevated levels of liver enzymes compared to patients with benign tumors (ALT 39.9 vs 24.4, p = 0.2 and AST 44.9 vs 23.4, p = 0.01), a trend also observed in CCA patients (ALT 45.2 vs 24.4, p = 0.06 and AST 46.5 vs 26.5, p < 0.05).

**Table 1:** Patient demographic information.

	Healthy (n = 19)	Benign (n = 23)	HCC (n = 19)	CRLM (n = 40)	CCA (n = 22)	NET (n = 10)
TNM Stage (stage: number of patients)	NA	NA	1:(5/19) 2: (10/19) 3: (4/19)	All Stage 4	1: (4/22) 2: (9/22) 3: (2/22) 4: (2/22)	All Stage 4
Grade (grade: number of patients)	NA	NA	1: (1/19) 2: (13/19) 3: (2/19) Unknown: (3/19)	NA	1: (2/22) 2: (6/22) 3: (8/22) Unknown: (6/22)	1: (3/10) 2: (4/10) 3: (3/10)
Liver cirrhosis	NA	1/23	9/19	0/18	4/22	0/10
Number of hepatic lesions: median (range)	NA	2 (1–10)	1 (1–3)	3 (1–13)	1 (1–5)	1 (1–7)
Size of largest lesion diameter in cm: median (range)	NA	3.6 (1.4–20)	4 (2.4–14.7)	2.5 (0.4–9.5)	4.6 (1–13.3)	2.4 (0.6–14.8)
Vascular invasion	NA	NA	11/19	4/40	10/22	5/10
Age [mean (range)]	46 (22–66)	47.6 (19–80)	71.4 (54–87)	55 (26–84)	67 (33–86)	55.9 (29–67)
Sex (% male)	21%	9%	47%	43%	50%	40%
ALT (Mean: St Dev)	NA	24.4 (± 34.7)	39.9 (± 33.2)	27.5 (± 17)	45.2 (± 38.5)	38.0 (± 14.7)
AST (Mean: St Dev)	NA	26.5 (± 22.3)	44.9 (± 20.3)	28.6 (± 13.2)	46.5 (± 30.9)	42.8 (± 38.1)
Albumin (Mean: St Dev)	NA	4.3 (± 0.4)	4.0 (± 0.5)	4.1 (± 0.4)	4.0 (± 0.5)	4.2 (± 0.3)
WBC (Mean: St Dev)	NA	7.0 (± 2.1)	7.7 (± 2.4)	6.6 (± 2.0)	7.0 (± 2.1)	7.9 (± 1.0)
Platelet (Mean: St Dev)	NA	253 (± 77)	208 (± 104)	192 (± 75)	241 (± 123)	275 (± 75)

### MDSCs are increased in the peripheral blood of patients with primary and metastatic liver tumors

**Figure 1:**
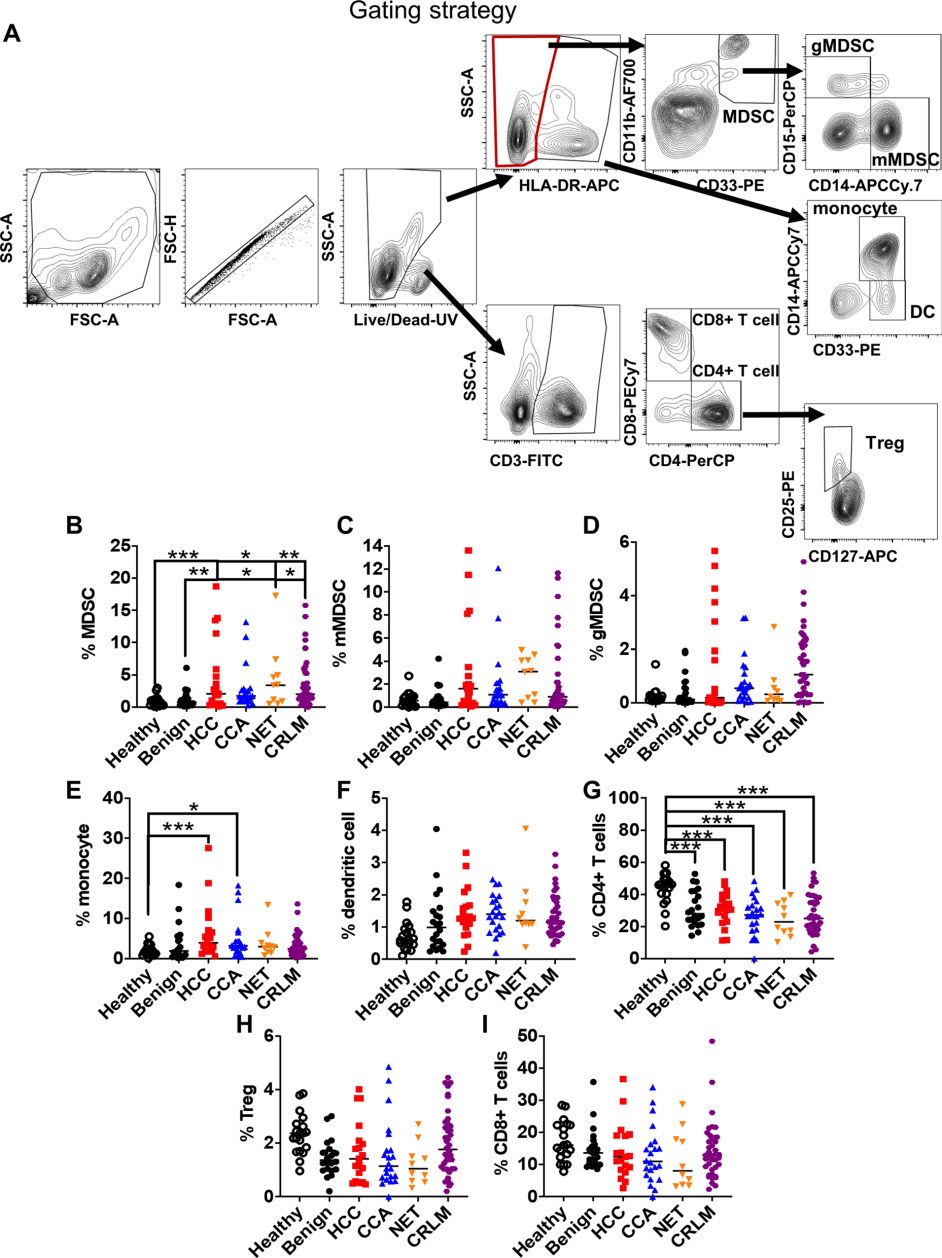
The frequency of MDSCs is increased in patients with HCC, CRLM and NET. (**A**) Representative gating strategy for the analysis of immune cell populations from peripheral blood. The frequency of (**B**) MDSCs (CD33^+^CD11b^+^HLA-DR^−/low^), (**C**) mMDSCs (CD33^+^CD11b^+^HLA-DR^−/low^CD14^+^), (**D**) gMDSCs (CD33^+^CD11b^+^HLA-DR^-/low^CD14^−^), (**E**) monocytes (CD33^+^CD11b^+^HLA-DR^+^CD14^+^), (**F**) dendritic cells (CD33^+^CD11b^+^HLA-DR^+^CD14^−^), (**G**) CD4^+^ T cells (CD3^+^CD4^+^CD8^−^) (**H**) Tregs (CD3^+^CD4^+^CD25^+^CD127^−^), and (**I**) CD8^+^ T cells (CD3^+^CD4^−^CD8^+^) in PBMCs of patients with CRLM (n = 41), HCC (n = 18), CCA (n = 18), NET (n = 5) and benign lesions (n = 18) and in healthy donors (n = 19) was analyzed with flow cytometry. *p < 0.05; **p < 0.01; ***p < 0.001 as determined for the myeloid and lymphoid populations by two-way ANOVA corrected for multiple comparisons with Tukey’s test. Only significant results are shown.

MDSCs are bone marrow-derived immature immunosuppressive cells that accumulate in patients with malignancies^9,17^. In humans, MDSCs are broadly categorized into monocytic (mMDSCs, CD33^+^CD11b^+^HLA-DR^-/low^CD14^+^) and granulocytic (gMDSC, CD33^+^CD11b^+^HLA-DR^-/low^CD14^-^) subsets that differ in their functional activity^7,18^. Previous studies demonstrated that mMDSCs are increased in the circulation of HCC patients and that their levels are impacted by radiation therapy^13–16^. To determine whether an increased frequency of MDSCs is observed in the circulation across different hepatobiliary malignancies, we investigated the frequency of these cells using the gating strategy provided in Fig. 1A. Consistent with earlier reports, the frequency of CD33^+^CD11b^+^HLA-DR^−/low^ MDSCs was elevated in HCC patients compared to patients with benign liver lesions and healthy donors (Fig. 1B). Additionally, there was a trend toward increased MDSC abundance with tumor grade in HCC patients (Fig. 1B, Supplementary Fig. 1A). A similar increase in the circulating bulk MDSC frequency, which was not driven by a particular subset, was observed in patients with NET and CRLM (Fig. 1B–D). In contrast, overall MDSC percentages in CCA patients were indistinguishable from those of patients with benign liver lesions independent of tumor grade (Fig. 1B–D, Supplementary Fig. 1B). Prior studies suggest that mMDSCs comprise the main population of MDSCs in patients with liver cancers^14–16^. Our findings demonstrated that CD14^+^CD15^−^ mMDSCs constitute the majority of MDSCs in the peripheral circulation, with, on average, a 3–20-fold difference in the representation of this subset compared to gMDSCs in patients and healthy donors (Supplementary Fig. 1C). Although the increase in individual MDSC subsets was limited (Fig. 1C,D), we detected a skewed mMDSC/gMDSC ratio in patients with HCC compared to healthy donors and CCA patients, indicative of an enhanced representation of the mMDSC subtype (Supplementary Fig. 1C). Previous studies also demonstrated that increased monocyte counts can associate with HCC recurrence and CCA invasiveness^19,20^. Consistently, there was an increase in monocyte frequency in both patient subpopulations, with no change in the percentage of dendritic cells (Fig. 1E,F).

Regulatory T cells (Tregs) constitute another major immunosuppressive cell population that suppresses the activity of anti-tumoral CD8^+^ T cells in the tumor microenvironment and is frequently increased in patients with cancer^21^. It has been established that MDSCs and Tregs cross-talk to induce the generation and recruitment of one another^22^. We analyzed the frequency of total CD4^+^ T cells, Tregs and CD8^+^ T cells to investigate whether these populations correlate with disease status. Compared to healthy donors, there was a decrease in total CD4^+^ T cells in all patient subpopulations (Fig. 1G). In contrast, neither Tregs nor activated CD8^+^ T cells were significantly different among various types of primary and metastatic liver malignancies or healthy donors (Fig. 1H,I). Together, these results indicate that MDSCs are the primary cell population that is enhanced in malignant liver tumors; however, MDSC frequency in patients with CCA was indistinguishable from that of patients with benign lesions.

To investigate whether MDSC frequency correlates with clinical parameters, our patient cohort was split into MDSC-high (n = 23) and MDSC-low (n = 86) groups based on the mean peripheral MDSC percentage (4.5%) in the HCC subgroup. There was no significant difference between AST (38.3. vs 34.7, p = 0.5) and ALT (36.6 vs 32.6, p = 0.6) between MDSC-high versus MDSC-low groups (Supplementary Fig. 2A). Next, we examined whether MDSC frequency was linked to tumor markers in HCC and CRLM. There was no significant correlation between MDSC frequency and AFP levels in patients with HCC (p = 0.09), but high MDSC levels (> 4.5%) correlated with elevated CEA in CRLM patients (Supplementary Fig. 2B,c).

### Malignant liver tumors have a distinct myeloid cell infiltration pattern compared to benign lesions

**Figure 2:**
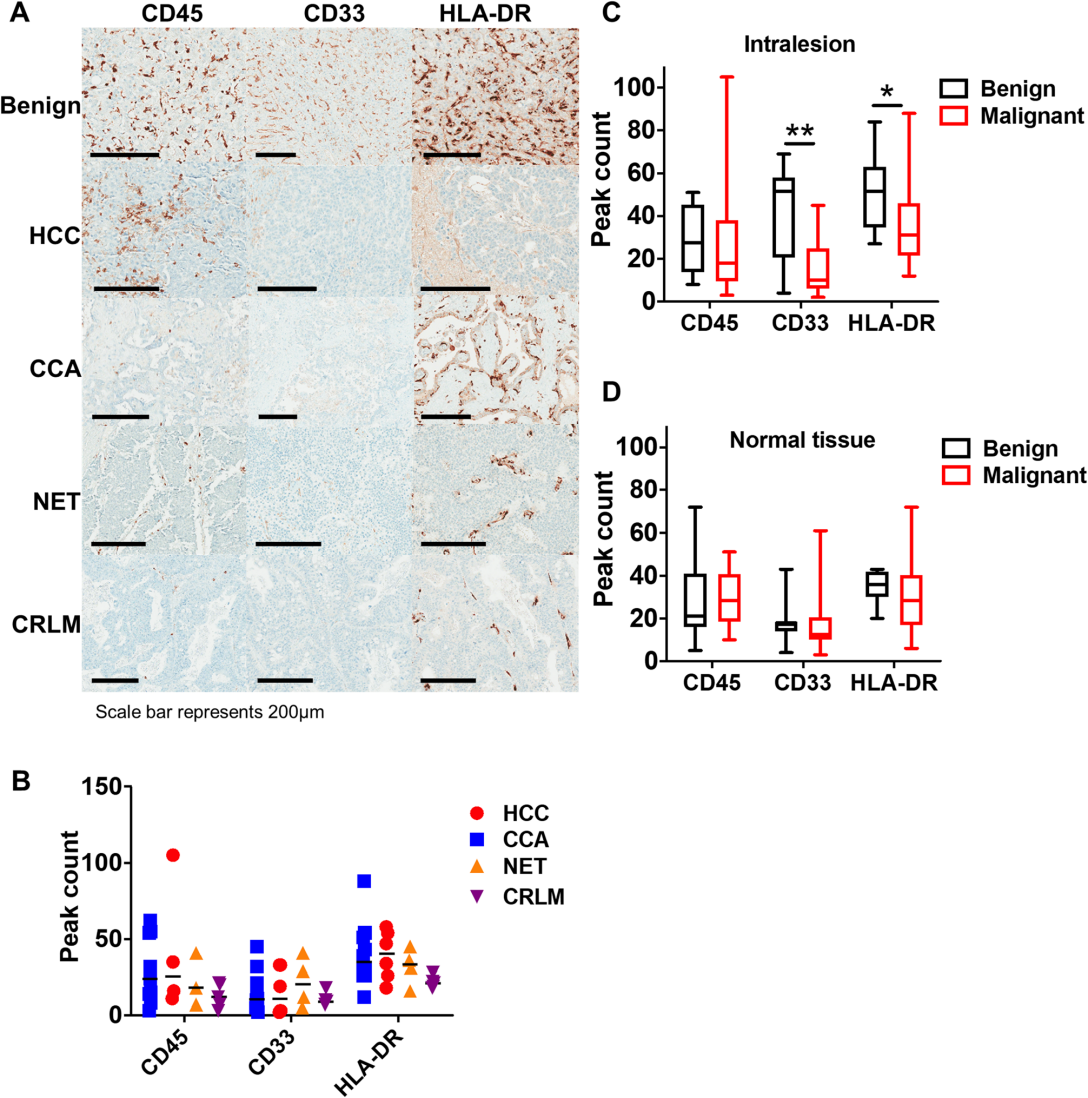
Malignant tumors have a distinct myeloid cell infiltration pattern compared to benign liver lesions. (**A**) Representative immunohistochemistry images demonstrating infiltration of CD45^+^, CD33^+^ and HLA-DR^+^ cells within the liver lesions. Scale bar in bottom left represents 200 μm. (**B**) Quantification of intralesional CD45, CD33 and HLA-DR peak area count from HCC (n = 6), CCA (n = 10), NET (n = 4) and CRLM (n = 5) cases. Quantification of CD45, CD33 and HLA-DR peak area count from (**C**) lesions (n = 10 benign versus n = 21–25 malignant) and (**D**) adjacent normal tissue (n = 7 benign versus n = 20 malignant). Data shown as box and whiskers from min to max with median. *p < 0.05, **p < 0.01 as determined by two-way ANOVA corrected for multiple comparisons with Tukey’s test.

Based on the observation that MDSCs and monocytes are differentially increased in the circulation of patients with hepatobiliary malignancies, we analyzed the innate immune cell infiltration pattern via immunohistochemistry staining of CD45, the pan-myeloid marker CD33 and MHC Class II (HLA-DR) in a subset of patients with HCC, CCA, NET, CRLM and benign lesions (Fig. 2A). Malignant tumors exhibited a similar intralesional immune profile with no significant differences in the number of CD45^+^, CD33^+^ and HLA-DR^+^ cells in peak areas within the tumors (Fig. 2B). Therefore, we focused on the differences between malignant and benign lesions. CD45 peak counts were similar across malignant and benign lesions. However, compared to the malignant tumors, benign lesions had higher numbers of intralesional CD33^+^ or HLA-DR^+^cells (15.44 CD33^+^ cells in malignant vs 40.90 in benign lesions, p < 0.01; 34.92 HLA-DR^+^ cells in HCC-CCA vs 52.10 in benign lesions, p < 0.05; Fig. 2C), indicating an increase in antigen-presenting cells (APCs). Normal tissue surrounding the lesions had similar numbers of CD45^+^ , CD33^+^ and HLA-DR^+^ cells, suggesting that the observed differences are driven by the tumors (Fig. 2D).

### MDSC frequency correlates with HCC outcome

**Figure 3:**
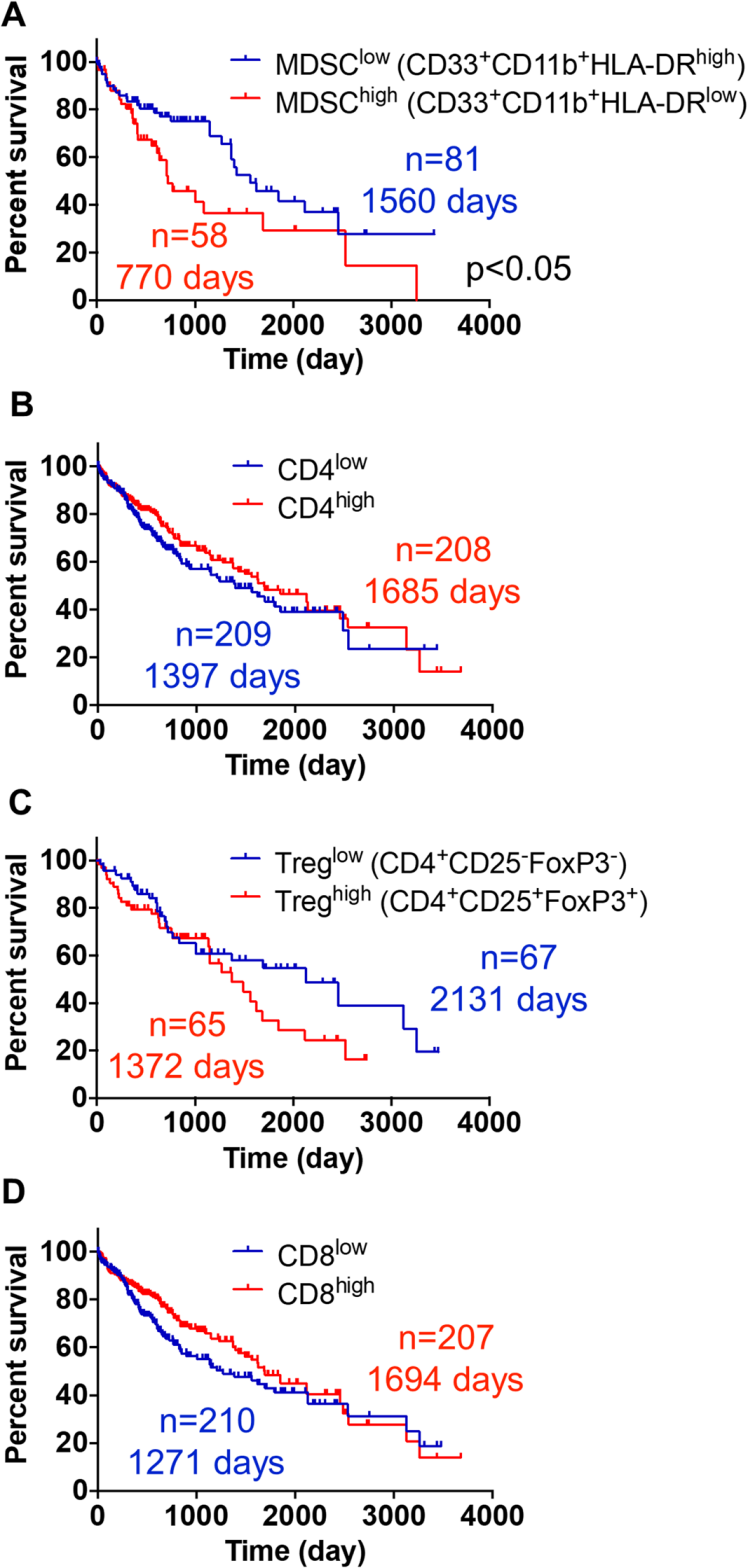
High MDSC score predicts poor HCC prognosis. Kaplan–Meier curves depicting overall survival of HCC patients with (**A**) a high (CD33^+^CD11b^+^HLA-DR^−/low^, n = 58, median survival 770 days) versus low (CD33^+^CD11b^+^HLA-DR^+^, n = 81, median survival 1560 days) MDSC score, (**B**) a high (n = 208, median survival 1685 days) versus low (n = 209, median survival 1397 days) CD4^+^ T cell score, (**C**) a high (CD4^+^CD25^+^FoxP3^+^, n = 65, median survival 1372 days) versus low (CD4^+^CD25^−^FoxP3^−^, n = 67, median survival 2131 days) Treg score, and (**D**) a high (n = 207, median survival 1694 days) versus low (n = 210, median survival 1271 days) CD8^+^ T cell score. Data are from TCGA LIHC database and were obtained from the UCSC Xena Browser. p < 0.05 was determined by log-rank (Mantel-Cox) test.

Although HCC patients had significantly higher levels of MDSCs compared to patients with benign lesions, HCC patients clustered into two groups based on levels of MDSC in PBMCs [MDSC high (> 4.5%) and low (< 4.5%)] (Fig. 1B). Based on this variation within PBMCs, we investigated whether MDSC frequency within the tumor correlates with disease outcome using the TCGA dataset containing gene expression information for 440 HCC patients. We generated a high versus low MDSC score by focusing on patients with high CD33 and CD11b expression and dividing these patients into to subgroups based on the median levels of HLA-DR expression. Patients with a high MDSC score (CD33^high^CD11b^high^HLA-DR^low^) had significantly reduced overall survival duration compared to those with a low MDSC score (CD33^high^CD11b^high^HLA-DR^high^; Fig. 3A). Importantly, this survival difference was not skewed by tumor grade, viral status or biological sex, as no significant differences for these parameters were detected between cohorts (data not shown). Due to the observed reduction in the frequency of CD4^+^ T cells in patients with hepatobiliary malignancies, we investigated the prognostic role of T cell infiltration (Fig. 1G). Neither CD4^+^ , CD8^+^ T, nor Treg cell signatures correlated with the survival of HCC patients (Fig. 3B–D).

## Discussion

MDSCs have been linked to suppression of the anti-tumor immune response and are emerging targets of cancer immunotherapy. Previous studies have demonstrated that the frequency of MDSCs in the peripheral blood is increased in various malignancies, including HCC^13–17^, which can impair therapeutic response^9,10^. Here, we demonstrated that different types of liver malignancies have distinct MDSC profiles. Despite increased MDSCs in the blood of patients with HCC, CRLM and NET, MDSC levels remained unaltered in patients with CCA compared to either those with benign lesions or healthy controls. CCA is broadly categorized as intrahepatic or extrahepatic, which vary in symptoms, prognosis and treatment response^23^. In our cohort, all but one patient with CCA had the intrahepatic subtype, preventing assessment of any potential correlations between CCA localization and MDSC accumulation pattern. Though the molecular mechanisms underlying this differential MDSC accumulation pattern in CCA remain to be investigated, the distinct mutational profile of liver malignancies may provide a explanation. The enzymes isocitrate dehydrogenase (IDH) 1/2, which are more frequently mutated in CCA compared to HCC, have been shown to alter the immune profile in other cancers^24,25^. Consistently, we observed that D-2-hydroxyglutarate (D2HG), which is produced by the mutant IDH1/2, limits MDSC generation (data not shown), suggesting that hepatobiliary malignancies can employ distinct mechanisms to regulate immune profile.

MDSCs subsets differ in their suppressive activity, ability to infiltrate tumors and disease associations. A recent study using a breast cancer model demonstrated that mMDSCs can modulate the local tumor microenvironment by interacting with cancer stem cells, while gMDSCs promote lung metastasis^18^. Consistent with previous observations^13–16^, our results demonstrated that the mMDSC subset (CD14^+^/CD15^−^/HLA-DR^−^) constitutes the majority of MDSCs detected in the circulation of patients with primary or metastatic liver tumors and that there is an approximately twofold increase in the frequency of these cells in patients with HCC. Although gMDSCs represented the minority of MDSCs, these cells can be affected by sample storage conditions^26^, and further analysis is required to determine whether the frequency of this population is differentially augmented in hepatobiliary malignancies and across tumor grades. Additionally, another population of MDSCs commonly referred to as early-stage MDSCs (e-MDSCs) were not investigated due to their unclear functional significance. In addition to the loss of statistical power, this population of MDSCs may explain why we saw no significant differences when we compared MDSC subsets to controls. It is important to note that we found no correlation between MDSC percentages and other clinical parameters such as TNM stage, AST, ALT or tumor marker levels. This indicates that MDSC frequency is an independent predictor of malignancy and tumor myeloid cell infiltration profile and is not confounded by clinical variables in HCC^27^.

One of the MDSC-dependent immunosuppressive mechanisms is induction of Tregs^22^. Kalathil et al. demonstrated that compared to healthy controls, the frequency of FoxP3^+^CTLA4^+^ Tregs was increased in HCC patients^13^. However, we did not observe a difference in the peripheral blood levels of CD25^+^CD127^−^ Tregs in patients with malignant liver tumors. While FoxP3 is widely used as a biomarker of Tregs, a lack of CD127 expression more reliably marks activated Tregs^28^. Additionally, our patient cohort excludes any viral HCC cases. HBV/HCV infection has been shown to recruit Tregs to restrain anti-viral immunity and inflammation-related liver damage^29,30^. Therefore, it is possible that patients with viral HCC have more Tregs than patients with non-viral HCC. While 75% of HCC cases result from chronic HBV/HCV infection, non-viral HCC patients are diagnosed with higher tumor burden, indicating the importance of considering HCC etiology^31^. While we observed a reduction in CD4^+^ T cells compared to healthy donors, this was not specific to any particular disease and therefore likely not linked to MDSCs. As peripheral blood was collected from patients during surgical resection, it is possible that the injury response may have induced changes in the immune profile, supporting the use of samples from patients with benign conditions who underwent surgery as a control. In line with this observation, HCC patients with a high MDSC (CD33^high^CD11b^high^HLA-DR^low^) but not CD4^+^ T cell or Treg score in the TCGA dataset had a survival disadvantage.

Unlike gMDSCs, which are hypothesized to be terminally differentiated, mMDSCs can polarize into APCs^32,33^. As mMDSCs were the prevalent cell type in patients with liver cancer, we speculated that the mMDSC-APC differentiation axis was hindered, leading to accumulation of mMDSCs in tissues. The lower number of intralesional CD33^+^ cells in malignant tumors points to a potential reduction in myeloid cell infiltration. It is also possible that the CD33 staining intensity biased the results, as APCs express higher levels of CD33 compared to immature myeloid cells^34^. In contrast, the decreased number of HLA-DR^+^ cells in malignant tumors compared to benign lesions may suggest a reduced ability to elicit an immune response in these tumors. One possible explanation for the decreased CD33^+^ and HLA-DR^+^ cells in malignant tumors is that ENTPD2/CD39L expression by tumor cells hinders MDSC maturation in liver tumors^35^. Although this has yet to be confirmed in HCC patients, the low MDSC and high monocyte frequencies in patients with CCA suggest that there might be alternative mechanisms that impair trafficking or maturation of APCs in this tumor type.

**Figure 4:**
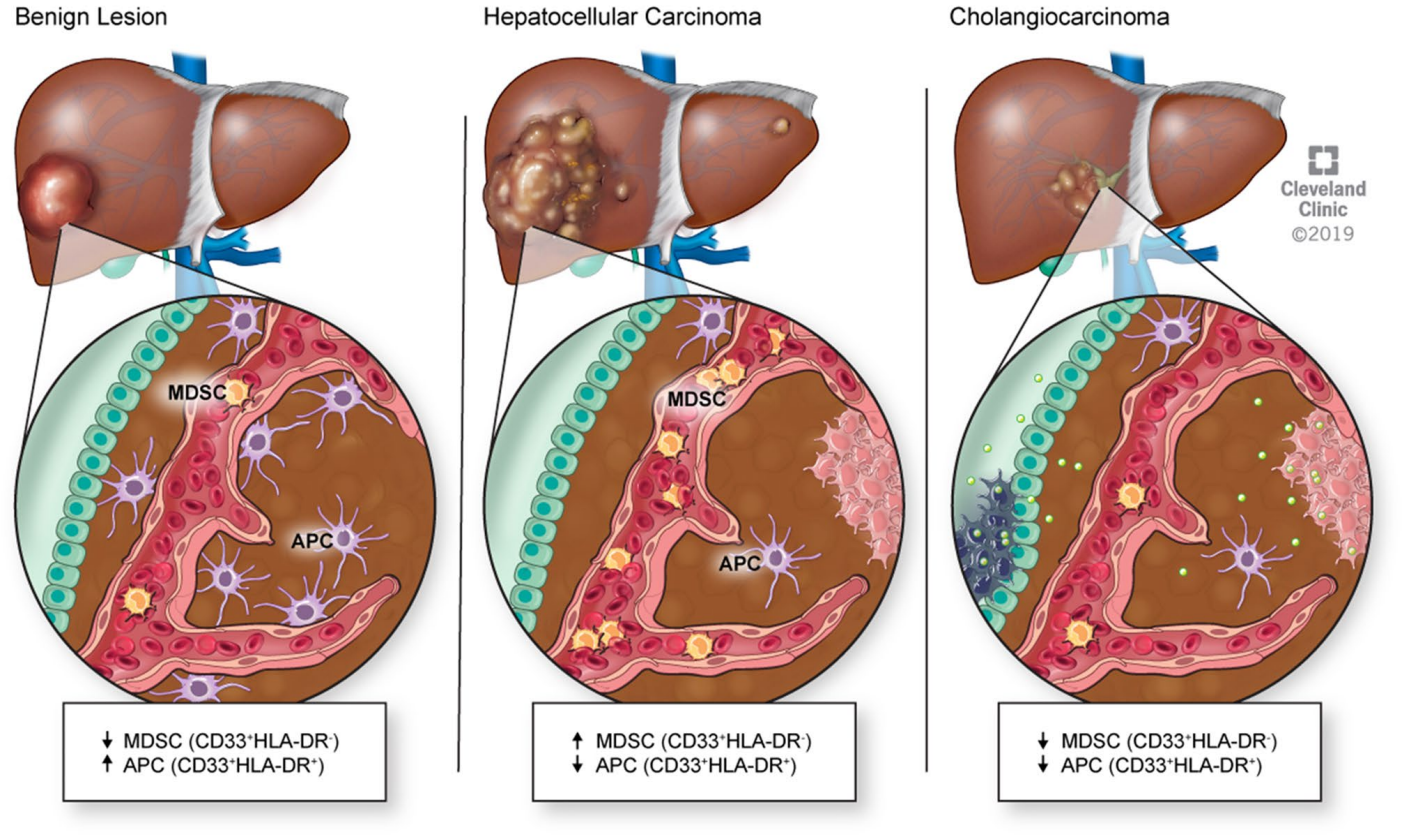
Malignant liver cancers have more circulating MDSCs and less tumor-infiltrating APCs.

Collectively, our results identify a distinct immune signature presented as high circulating MDSC levels and low tumor-infiltrating APCs in non-viral HCC as opposed to low peripheral MDSCs and low tumor-infiltrating APCs in CCA and low circulating MDSCs and increased intrahepatic APCs in benign lesions (Fig. 4). Our findings make the case for further studies investigating a role for blood MDSC measurements as a diagnostic test for primary and metastatic hepatobiliary malignancies and a potential role for therapeutic targeting. The use of metronomic chemotherapy to deplete MDSCs^11,36–39^ is currently under clinical evaluation for glioblastoma and non-small cell lung cancer (NCT02669173, NCT03302247). This approach represents a promising therapeutic opportunity for HCC, CRLM and NETs.

## Materials and methods

### Reagents

Fluorophore-conjugated anti-human CD3 (UCHT1), CD4 (SK3), CD8 (RPA-T8), CD14 (MφP9), CD25 (M-A251), CD33 (WM53), CD127 (HIL-7R-M21) and HLA-DR (G46-6) antibodies were purchased from BD Biosciences. CD15 (HI98, BD Bioscience) was conjugated with PerCP using the Expedeon Lightning-Link kit. Anti-human CD11b (CD11B29) antibody and LIVE/DEAD Fixable Blue Dead Cell Stain Kit for UV excitation were obtained from ThermoFisher Scientific.

### Patients

Peripheral blood samples were collected perioperatively from 114 patients with non-viral liver cancer in the Cleveland Clinic. All patients provided written informed consent under IRB 10-347, and the protocol was approved by the Cleveland Clinic Institutional Review Board. All the diagnoses were confirmed by the Cleveland Clinic Pathology Department. Healthy donor specimens (n = 19) were acquired through the P50 Northern Ohio Alcohol Center (NOAC). All studies were performed in accordance with relevant guidelines and regulations. Patient clinical characteristics and donor demographical information are provided in Table 1.

### Quantification of MDSCs in the peripheral blood

Peripheral blood samples were processed using a Ficoll-Paque PLUS gradient in a SepMate (Stem Cell Technologies) and frozen in 10% DMSO (Fisher Scientific) and 90% FBS (Life Technologies) until further analysis. Samples were stained with live/dead UV stain (Life Technologies) and blocked in FACS buffer (PBS, 2% BSA) containing an FcR blocking reagent (Miltenyi Biotec). Samples were stained with fluorophore-conjugated antibody cocktails, washed with FACS buffer and analyzed using an LSRFortessa (BD Biosciences). Analyses were performed using FlowJo software (Tree Star Inc.). MDSC subset gating was done using established phenotypic guidelines^40^.

### Immunohistochemistry (IHC)

Formalin fixed paraffin-embedded blocks and H&E-stained slides from liver biopsy specimens were available for 29 patients (6 HCC, 10 benign, 10 CCA, 4 NET and 5 CRLM) for IHC analysis. IHC was performed using the mouse monoclonal CD33 antibody (PWS44, Leica Biosystems) diluted 1:100 using SignalStain Diluent (Cell Signaling Technology) and incubated for 40 min at 37 °C. Automated staining used the VENTANA BenchMark Ultra platform and the U iView DAB software and was detected using iView DAB with the Endogenous Biotin Blocking Kit (Ventana Medical Systems Inc.). Antigen retrieval used Cell Conditioning #1 selected for the standard time. The mouse monoclonal antibody HLA-DR/DP/DQ (clone CR3/43, ThermoFisher) used the VENTANA UltraView DAB detection kit and U ultraView DAB software with the BenchMark Ultra automated stainer. The dilution used was 1:100 in SignalStain diluent, and samples were incubated for 40 min at 37 °C. Antigen retrieval used Cell Conditioning #1 selected for the standard time. Mouse antibody amplification was selected. CD45 antibody (2B11 PD7/26, Dako) was diluted 1:100 and incubated for 16 min at 37 °C. Ventana optiView DAB with pre-primary peroxidase inhibitor selection was used for detection. Normal lymph node tissue or tonsils were used for positive run controls. IHC staining conditions are summarized in Supplementary Table 1.

HLA-DR^+^, CD33^+^ and CD45^+^ cells were assessed quantitatively within the intralesional and tumor-adjacent normal adjacent tissue at a 200× magnification on an Olympus B41 microscope. For all markers, the peak count was considered.

### Bioinformatic analysis of immune populations

The Cancer Genome Atlas (TCGA) Liver Hepatocellular Carcinoma (LIHC) database was accessed via the University of California Santa Cruz (UCSC) Xena Browser (https://xenabrowser.net/heatmap/) on March 21, 2018; December 13, 2018; and March 26, 2020. CD33, CD11b (ITGAM), HLA-DRA, CD4 and CD8a expression levels; patient demographic information; viral status; and overall survival data were extracted for further analysis. To generate the MDSC signature, only samples with CD33 and CD11b expression above the median values were used. From this set, samples with low HLA-DRA expression were categorized into the “high MDSC signature” group. CD33^high^CD11b^high^HLA-DRA^high^ samples were used for the control population. Treg signature was generated based on the initial CD4^+^ gating and subsequent high CD25 and FoxP3 expression levels. Samples with CD25 and FoxP3 levels below the median served as controls. Median CD4 and CD8 expression values were used to discriminate patients with high versus low T cell infiltration.

### Statistical analysis

To test differences in continuous variables with normal distribution, a two-way ANOVA was performed with Tukey’s test for multiple comparisons. The long-rank test was utilized to determine differences in overall survival. p-values < 0.05 were considered statistically significant (GraphPad Software Inc., JMP Pro Version 14. SAS Institute Inc. Cary, NC, 1989–2020).

## Supplementary information

Supplementary Information.

## Data Availability

Raw data will be made available upon request from the corresponding author.

## Abbreviations

AFP: Alpha-fetoprotein
AST: Aspartate aminotransferase
ALT: Alanine transaminase
APC: Antigen-presenting cell
CCA: Cholangiocarcinoma
CEA: Carcinoembryonic antigen
CRLM: Colorectal liver metastases
DC: Dendritic cell
gMDSC: Granulocytic myeloid-derived suppressor cell
HCC: Hepatocellular carcinoma
MDSC: Myeloid-derived suppressor cell
mMDSC: Monocytic myeloid-derived suppressor cell
NET: Neuroendocrine tumor
PBMC: Peripheral blood mononuclear cell
TCGA: The Cancer Genome Atlas
Treg: T regulatory cell
WBC: White blood cell
