# Supplementary material for: Hepatobiliary malignancies have distinct peripheral myeloid-derived suppressor cell signatures and tumor myeloid cell profiles
Source: Sci Rep. 2020 Nov 2;10:18848. doi: 10.1038/s41598-020-75881-1 (PMC7606602; doi:10.1038/s41598-020-75881-1)
Supplement: Supplementary file 1 — Supplementary Information. [file 41598_2020_75881_MOESM1_ESM.pptx]

## Slide 1
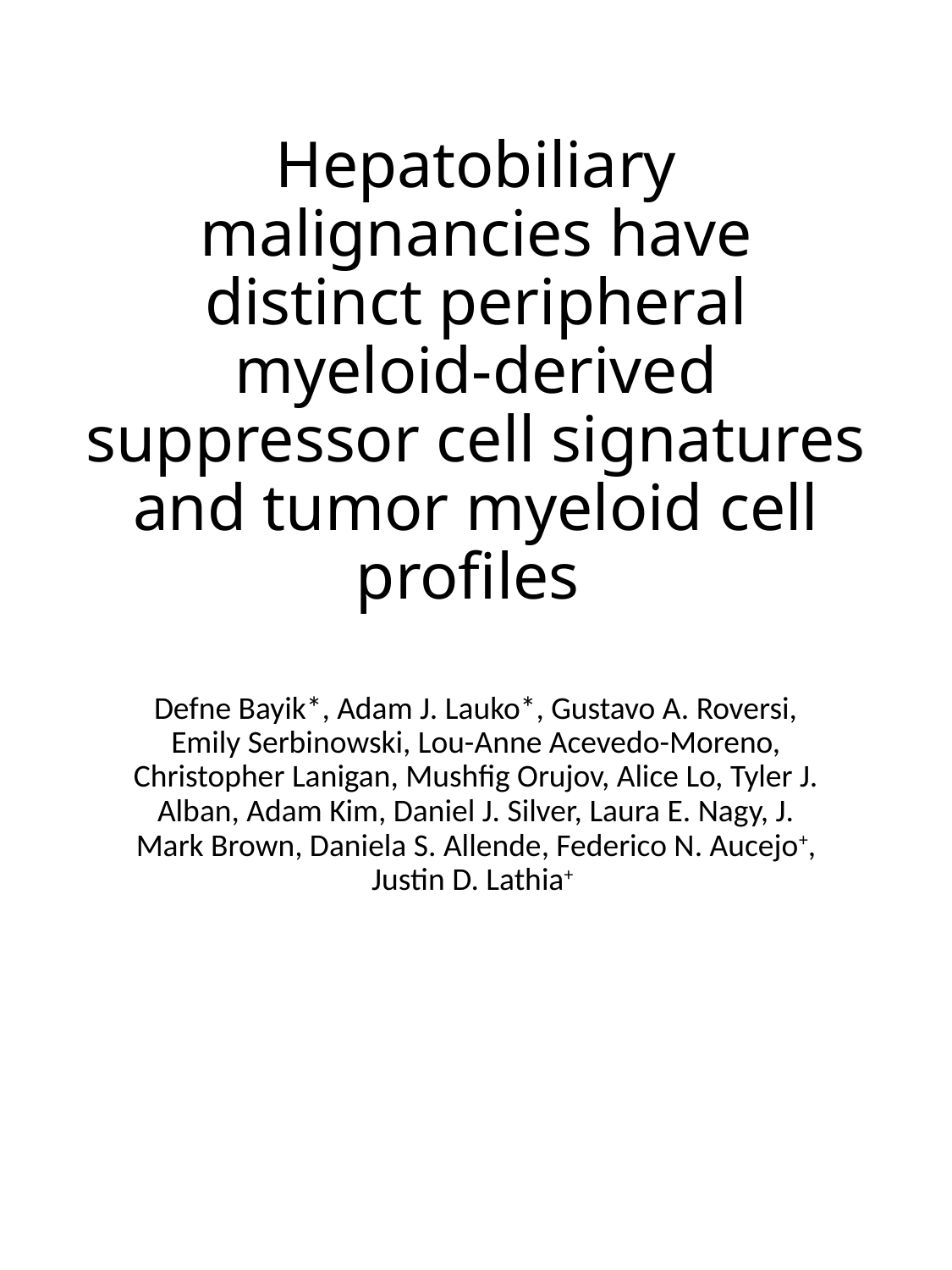

# Hepatobiliary malignancies have distinct peripheral myeloid-derived suppressor cell signatures and tumor myeloid cell profiles
Defne Bayik*, Adam J. Lauko*, Gustavo A. Roversi, Emily Serbinowski, Lou-Anne Acevedo-Moreno, Christopher Lanigan, Mushfig Orujov, Alice Lo, Tyler J. Alban, Adam Kim, Daniel J. Silver, Laura E. Nagy, J. Mark Brown, Daniela S. Allende, Federico N. Aucejo+, Justin D. Lathia+

## Slide 2
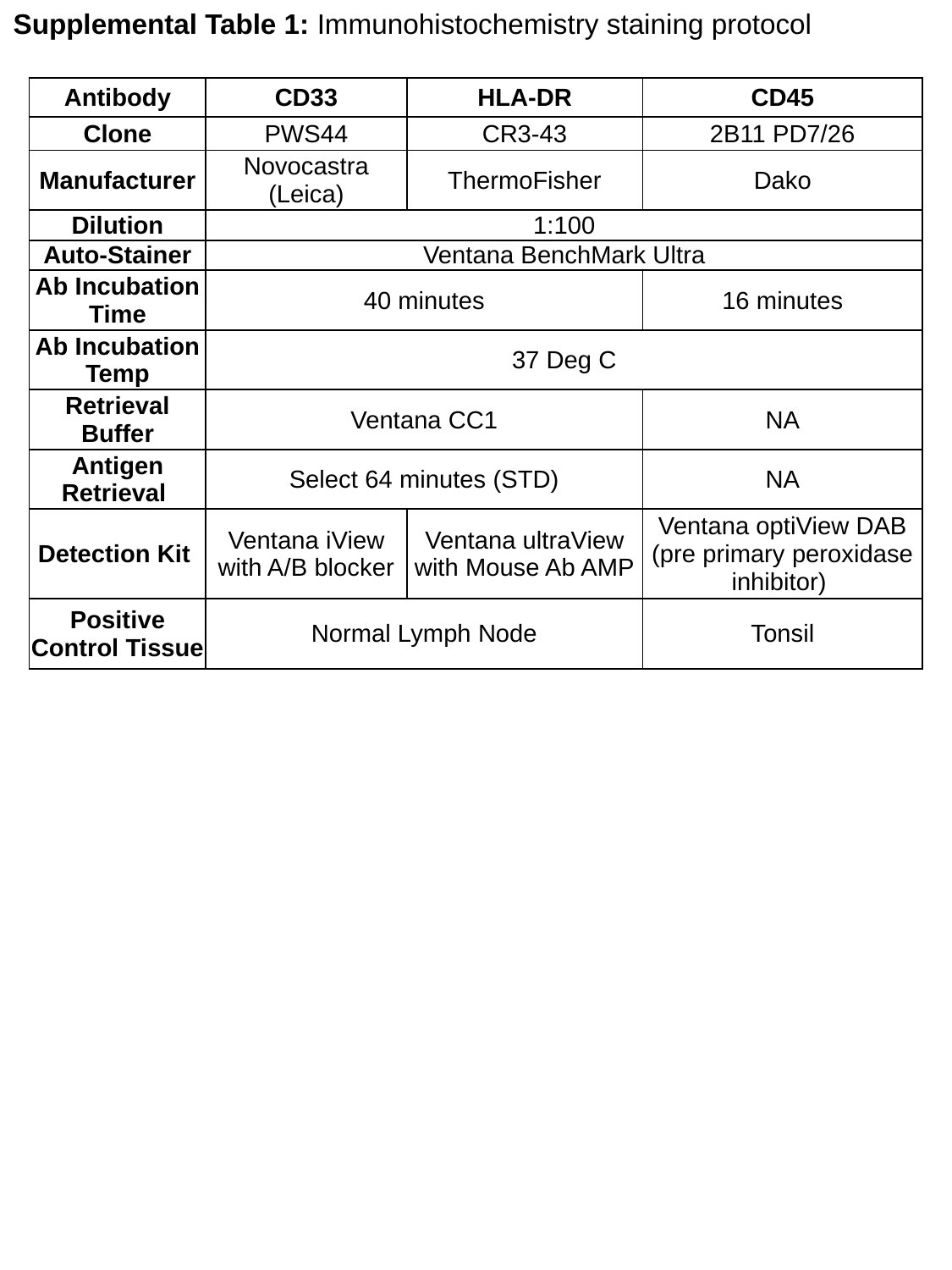

Supplemental Table 1: Immunohistochemistry staining protocol
| Antibody | CD33 | HLA-DR | CD45 |
| --- | --- | --- | --- |
| Clone | PWS44 | CR3-43 | 2B11 PD7/26 |
| Manufacturer | Novocastra (Leica) | ThermoFisher | Dako |
| Dilution | 1:100 | | |
| Auto-Stainer | Ventana BenchMark Ultra | | |
| Ab Incubation Time | 40 minutes | | 16 minutes |
| Ab Incubation Temp | 37 Deg C | | |
| Retrieval Buffer | Ventana CC1 | | NA |
| Antigen Retrieval | Select 64 minutes (STD) | | NA |
| Detection Kit | Ventana iView with A/B blocker | Ventana ultraView with Mouse Ab AMP | Ventana optiView DAB (pre primary peroxidase inhibitor) |
| Positive Control Tissue | Normal Lymph Node | | Tonsil |

## Slide 3
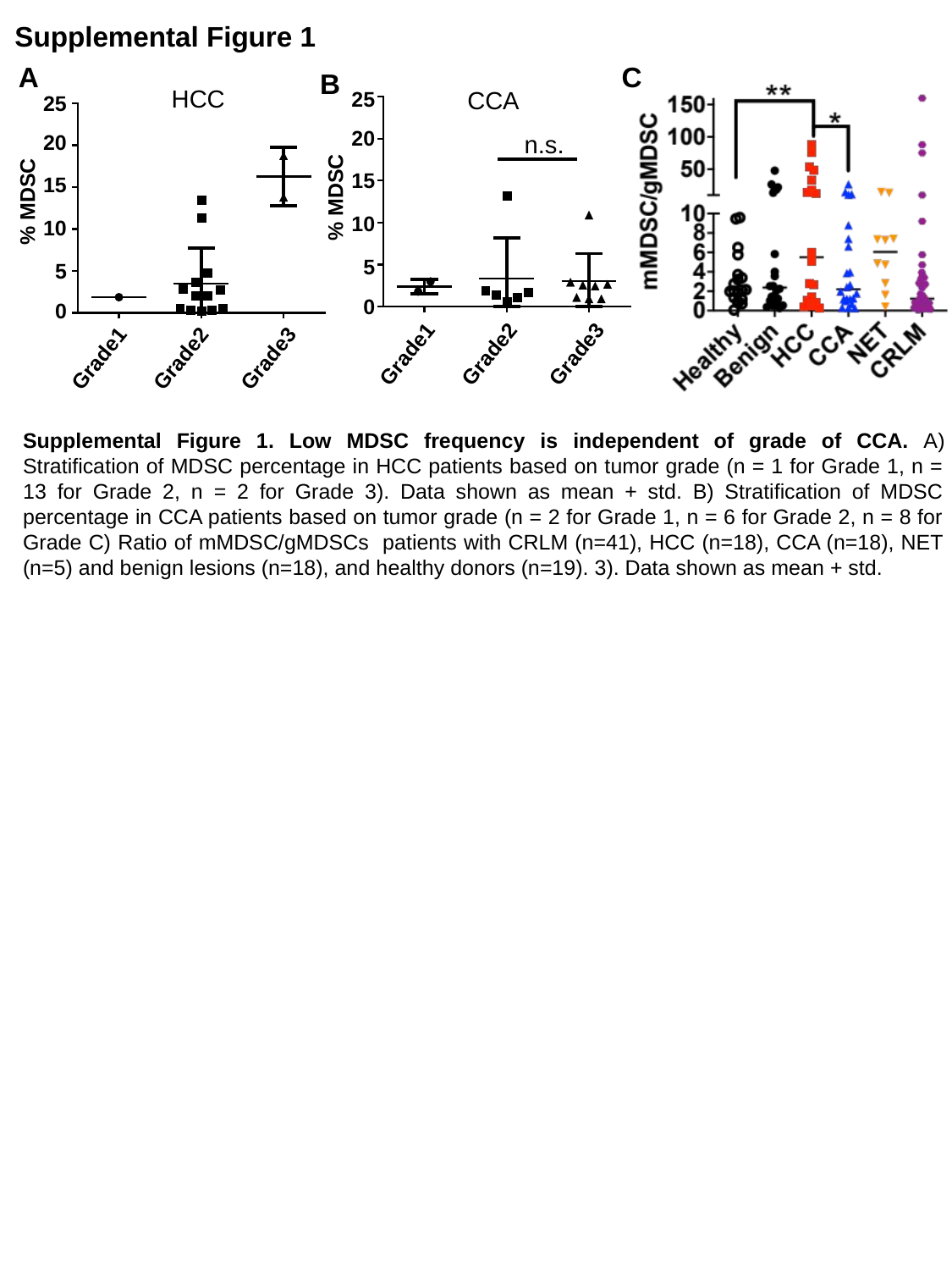

Supplemental Figure 1
A
C
B
CCA
25
n.s.
20
15
% MDSC
10
5
0
Grade1
Grade2
Grade3
HCC
25
20
15
% MDSC
10
5
0
Grade1
Grade2
Grade3
Supplemental Figure 1. Low MDSC frequency is independent of grade of CCA. A) Stratification of MDSC percentage in HCC patients based on tumor grade (n = 1 for Grade 1, n = 13 for Grade 2, n = 2 for Grade 3). Data shown as mean + std. B) Stratification of MDSC percentage in CCA patients based on tumor grade (n = 2 for Grade 1, n = 6 for Grade 2, n = 8 for Grade C) Ratio of mMDSC/gMDSCs patients with CRLM (n=41), HCC (n=18), CCA (n=18), NET (n=5) and benign lesions (n=18), and healthy donors (n=19). 3). Data shown as mean + std.

## Slide 4
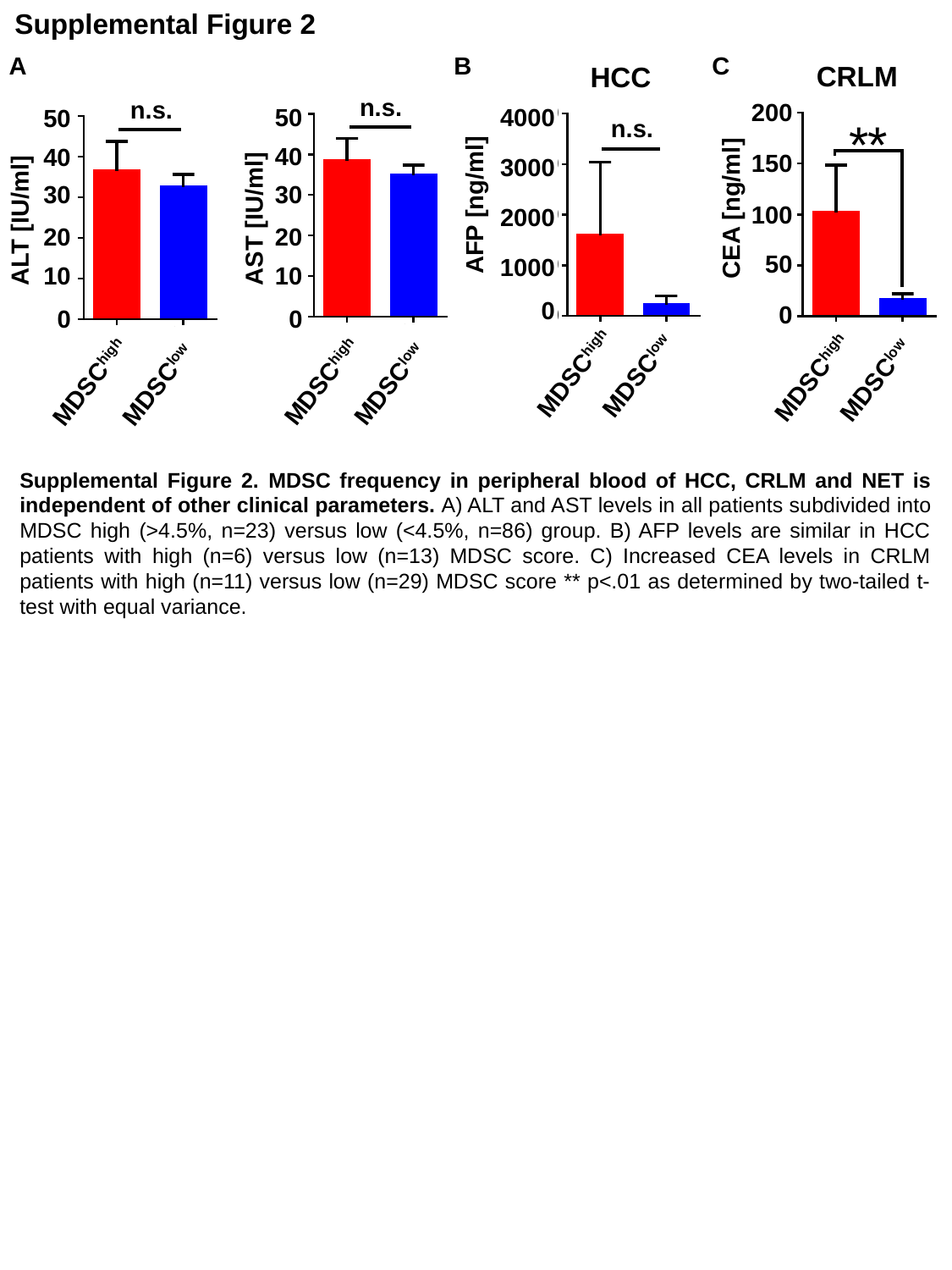

Supplemental Figure 2
A
B
C
CRLM
HCC
n.s.
n.s.
200
4000
50
50
n.s.
40
40
150
3000
30
30
AFP [ng/ml]
CEA [ng/ml]
AST [IU/ml]
ALT [IU/ml]
100
2000
20
20
50
1000
10
10
0
0
0
0
MDSChigh
MDSClow
MDSChigh
MDSClow
MDSChigh
MDSChigh
MDSClow
MDSClow
Supplemental Figure 2. MDSC frequency in peripheral blood of HCC, CRLM and NET is independent of other clinical parameters. A) ALT and AST levels in all patients subdivided into MDSC high (>4.5%, n=23) versus low (<4.5%, n=86) group. B) AFP levels are similar in HCC patients with high (n=6) versus low (n=13) MDSC score. C) Increased CEA levels in CRLM patients with high (n=11) versus low (n=29) MDSC score ** p<.01 as determined by two-tailed t-test with equal variance.
